# Cerebral μ-opioid and CB_1_ receptor systems have distinct roles in human feeding behavior

**DOI:** 10.1038/s41398-021-01559-5

**Published:** 2021-08-27

**Authors:** Tatu Kantonen, Tomi Karjalainen, Laura Pekkarinen, Janne Isojärvi, Kari Kalliokoski, Valtteri Kaasinen, Jussi Hirvonen, Pirjo Nuutila, Lauri Nummenmaa

**Affiliations:** 1 Turku PET Centre, University of Turku, Turku, Finland; 2 Clinical Neurosciences, University of Turku, Turku, Finland; 3 Department of Endocrinology, Turku University Hospital, Turku, Finland; 4 Neurocenter, Turku University Hospital, Turku, Finland; 5 Department of Radiology, University of Turku and Turku University Hospital, Turku, Finland; 6 Department of Psychology, University of Turku, Turku, Finland

**Keywords:** Molecular neuroscience, Physiology

## Abstract

Eating behavior varies greatly between individuals, but the neurobiological basis of these trait-like differences in feeding remains poorly understood. Central μ-opioid receptors (MOR) and cannabinoid CB_1_ receptors (CB_1_R) regulate energy balance via multiple neural pathways, promoting food intake and reward. Because obesity and eating disorders have been associated with alterations in the brain’s opioid and endocannabinoid signaling, the variation in MOR and CB_1_R system function could potentially underlie distinct eating behavior phenotypes. In this retrospective positron emission tomography (PET) study, we analyzed [^11^C]carfentanil PET scans of MORs from 92 healthy subjects (70 males and 22 females), and [^18^F]FMPEP-*d*_*2*_ scans of CB_1_Rs from 35 subjects (all males, all also included in the [^11^C]carfentanil sample). Eating styles were measured with the Dutch Eating Behavior Questionnaire (DEBQ). We found that lower cerebral MOR availability was associated with increased external eating—individuals with low MORs reported being more likely to eat in response to environment’s palatable food cues. CB_1_R availability was associated with multiple eating behavior traits. We conclude that although MORs and CB_1_Rs overlap anatomically in brain regions regulating food reward, they have distinct roles in mediating individual feeding patterns. Central MOR system might provide a pharmacological target for reducing individual’s excessive cue-reactive eating behavior.

## Introduction

Obesity is one of the leading public health issues, resulting from individuals’ long-term excessive energy intake in relation to energy expenditure [1]. Yet, humans vary greatly in their choices and habits related to food intake quantity and quality i.e., eating behavior [2, 3]. Trait-like eating behaviors have been associated with multiple clinical eating disorders in addition to obesity [4–7], but also nonobese individuals vary in how they control their feeding [8]. Interacting with peripheral hormones, central nervous system (CNS) integrates hunger and satiety signals with environmental stimuli to regulate food intake [1]. Large-scale genome-wide association studies have identified limbic system, hippocampus and hypothalamus to be key regions in the CNS contributing to individual’s body mass index (BMI) and eating behavior [9, 10]. Central regulation of feeding is however constantly challenged by the modern environment characterized by abundance of palatable and energy-dense food products, promoting feeding independently of metabolic needs [11, 12]. The prevalence of obesity is increasing in alarming speed, and new targets for anti-obesity pharmacotherapy are acutely needed [13].

Palatability and hedonic properties of food are centrally mediated by μ-opioid receptor (MOR) system [14, 15]. Both endogenous and exogenous opioids stimulate feeding, especially via hedonic hotspots of nucleus accumbens, insula and frontal cortex [16–19]. Conversely, opioid antagonists reduce food intake and related hedonic responses in rodents [19] and humans [20, 21]. Central MORs are also important mediators of homeostatic feeding, even in the absence of subjective pleasure [22]. Human positron emission tomography (PET) studies have revealed that obesity associates with decrease of MORs in appetite regulating brain areas [23, 24], and insular MORs are lowered in patients with bulimia nervosa proportionally to fasting behavior [25]. Central MOR system function varies considerably also in healthy humans [26], and traits linked with feeding control such as impulsivity are associated with MOR availability [27]. Nevertheless, the association between the MOR system and specific patterns of eating behavior remains elusive.

Feeding is also regulated by brain’s endocannabinoid system, which anatomically overlaps with MORs in the central reward circuit [28]. The most abundant central cannabinoid receptors are the CB_1_ receptors (CB_1_Rs), which regulate food intake through pathways of ventral striatum, limbic system, and hypothalamus [29, 30]. Functional interplay between MOR and CB_1_R systems has been established in animal studies, where CB_1_R-antagonists and MOR-antagonists have synergistic effect on reducing food intake [31], while CB_1_R-antagonist can be used to block MOR-agonist induced food intake and vice versa [32]. MOR-agonists also directly increase endocannabinoid concentration and CB_1_R-agonists increase opioid concentration in the brain [33, 34]. In humans with food intake disorders including obesity, anorexia and bulimia nervosa, lowered central CB_1_R availability in the mesolimbic reward system associates with increased BMI [35]. While CB_1_R-antagonist rimonabant showed promise as an anti-obesity drug, it had to be withdrawn due to psychiatric side effects [36]. More nuanced understanding of CB_1_R system and feeding is clearly required to enable further pharmacological advancement.

Variation in central MOR and CB_1_R function could thus be linked to differences in feeding behavior, but it remains unresolved what specific feeding traits they govern in humans. Individual differences in feeding can be conceptualized based on the psychological mechanisms that contribute to or attenuate development of overweight. In such conceptualization, *emotional eating* refers to reactive overeating to distress or negative emotions, while *external eating* refers to tendency to overeat in response to appetitive food-cues. Finally, *restrained eating* refers to the tendency to eat less than desired [37–39]. Variation in such trait-like feeding patterns contribute to differences in weight gain and maintenance [37, 40], and they can be measured using The Dutch Eating Behavior Questionnaire (DEBQ) [41]. In this retrospective study utilizing PET scans from historical healthy controls, we compiled 92 [^11^C]carfentanil scans of MOR system and 35 [^18^F]FMPEP-*d*_*2*_ scans of CB_1_R system and corresponding DEBQ scores. We tested whether the MOR and CB_1_R availabilities in food-intake-regulating brain areas associate with individual eating behavior traits measured with DEBQ.

## Materials and methods

### Subjects

The study subjects were historical controls retrieved from the AIVO neuroinformatics database (http://aivo.utu.fi), a large-scale molecular image database hosted by Turku PET Centre. We identified all the [^11^C]carfentanil and [^18^F]FMPEP-*d*_*2*_ baseline PET studies accompanied with completed Finnish version of the DEBQ form [41]. Exclusion criteria were neurologic and psychiatric disorders, current use of medications that could affect CNS or abuse of alcohol or illicit drugs. Subjects were not preselected on the basis of weight or eating habits. Final sample consisted of 92 subjects (70 males and 22 females) scanned with [^11^C]carfentanil from five distinct projects with three different PET scanners. The [^18^F]FMPEP-*d*_*2*_ sample consisted of 35 males, all of which were also all included in the [^11^C]carfentanil male sample. All subjects of the [^18^F]FMPEP-*d*_*2*_ subsample were nonsmoking males, while in the [^11^C]carfentanil sample seven females smoked. All [^18^F]FMPEP-*d*_*2*_ scans were carried out with GE Discovery VCT PET/CT (GE Healthcare). The original data were acquired between 2011 and 2019 in the Turku PET Centre (Turku, Finland). The subjects had completed the DEBQ form on the day of the scanning visit or on the preceding screening day. Characteristics of the study sample are summarized in Table 1, and the information of smoking status and PET scanners are detailed in Supplementary Table 1. The study was conducted in accordance with the Declaration of Helsinki and approved by the Turku University Hospital Clinical Research Services. The subjects had signed written informed consent forms and completed the DEBQ forms as a part of the original study protocols. The references for the original studies are provided in Supplementary Table 2. a priori power analysis based on our prior neuroreceptor PET studies on obesity [24] suggested that a sample size of 32 would be sufficient for establishing the predicted effects of *r* = 0.5 at power of 0.95.

**Table 1:** Characteristics of the studied subjects.

	[^11^C]carfentanil scans						*p* value
	Males (*n* = 70)			Females (*n* = 22)			
	Mean	SD	Range	Mean	SD	Range	
Age (years)	27.4	7.5	19–58	47.7	10.0	20–59	<0.001
BMI (kg/m^2^)	24.5	2.8	19–31	23.7	3.1	18–31	0.27
Total DEBQ score	67.0	12.8	40–109	73.4	12.8	46–97	0.05
Emotional eating score	21.0	6.8	13–40	22.1	5.7	13–32	0.44
External eating score	24.7	6.5	10–43	25.0	5.4	13–34	0.82
Restrained eating score	21.3	5.5	11–39	26.2	5.5	10–33	0.001
Injected activity (MBq)	277.0	77.9	223–508	352.3	125.5	234–519	0.01
	[^18^F]**FMPEP**-*d*_*2*_ **scans**						
	Males (*n* = 35)						
	Mean	SD	Range				
Age (years)	25.9	4.3	21–35				
BMI (kg/m^2^)	24.5	3.1	19–31				
Total DEBQ score	68.6	14.5	43–109				
Emotional eating score	20.7	7.4	13–40				
External eating score	27.1	6.0	14–43				
Restrained eating score	20.8	5.6	12–32				
Injected activity (MBq)	187.9	12.8	147–215				

*p* value is for two-tailed independent samples *t*-test between males and females.

### Eating behavior assessment with the DEBQ

The DEBQ [41] was used to quantify eating behavior. The DEBQ is a 33-item questionnaire with Likert-type scoring in each item (response options ranging from 1 to 5, from “Never” to “Very often”). It is divided in three dimensions measuring different behavioral traits: Emotional eating, External eating, and Restrained eating [37–39]. The emotional and external overeating are based on psychosomatic and externality theories of eating behavior, while restrained eating dimension centers around food intake self-inhibition [41]. The DEBQ subscales have been designed to measure independent dimensions of feeding behavior [42], and the subscales have good internal consistency, dimensional validity, and test-retest reliability [4, 7, 41, 43].

### Image processing and modeling

PET images were preprocessed similarly using automated processing pipeline Magia [44]. [^11^C]carfentanil data preprocessing has been described previously [26]. Briefly, preprocessing consisted of framewise realignment and coregistration of the PET and magnetic resonance images (MRIs). MOR availability was expressed as [^11^C]carfentanil binding potential (*BP*_ND_), which is the ratio of specifically bound radioligand to that of nondisplaceable radioligand in tissue [45]. *BP*_ND_ was estimated with simplified reference tissue model [46]. Occipital cortex served as the reference region, since it contains only negligible number of opioid receptors and yields reliable and reproducible reference estimates [47–49]. Parametric *BP*_ND_ images were calculated and spatially normalized to MNI-space via segmentation of T1-weighted MRIs and smoothed with an 8 mm Gaussian kernel. For [^18^F]FMPEP-*d*_*2*_, there exists no suitable central reference region (i.e., a region without CB_1_Rs)—thus, CB_1_R availability is expressed as the [^18^F]FMPEP-*d*_*2*_ volume of distribution (*V*_T_) [50]. [^18^F]FMPEP-*d*_*2*_ *V*_T_ was quantified using graphical analysis by Logan [51]. The frames starting at 36 min and later since injection were used in the model fitting, since Logan plots became linear after 36 min [51]. Plasma activities were corrected for plasma metabolites as described previously [52]. Further details of the scan acquisition and modeling of the [^18^F]FMPEP-*d*_*2*_ data are described in Supplementary Text 1.

### Statistical analysis

The study question was whether the DEBQ subscales (Emotional eating, External eating, Restrained eating) or Total DEBQ scores are associated with [^11^C]carfentanil *BP*_ND_ or [^18^F]FMPEP-*d*_*2*_ *V*_T_. We used Bayesian hierarchical modeling to analyze these effects in a priori regions of interest (ROIs). We targeted regions with high to moderate density of MORs [26, 53], and with previously proposed roles in obesity [24], food intake disorders [25, 54], and food reward [55–57]. The analyses were harmonized by investigating CB_1_Rs in the same regions. FreeSurfer (http://surfer.nmr.mgh.harvard.edu/) was used to extract the ten bilateral ROIs: amygdala, caudatus, cerebellum, dorsal anterior cingulate cortex, insula, middle temporal cortex, nucleus accumbens, orbitofrontal cortex, putamen, and thalamus. The Bayesian models were estimated using the R package brms (https://cran.r-project.org/web/packages/brms/index.html) that utilizes the Markov chain Monte Carlo sampling of RStan (https://mc-stan.org/users/interfaces/rstan). Because age influences [^11^C]carfentanil binding [26, 58] and different PET scanners may yield slightly different *BP*_ND_ estimates [59], both age and PET scanner were controlled for in all [^11^C]carfentanil models. Age was also controlled for in all [^18^F]FMPEP-*d*_*2*_ *V*_T_ models (the scanner-adjustment was not needed since the [^18^F]FMPEP-*d*_*2*_ data were acquired using a single scanner). For both tracers, we created models separately for the Total DEBQ score as well as its subscales, adjusting for age. [^11^C]carfentanil *BP*_ND_ and [^18^F]FMPEP-*d*_*2*_ *V*_T_ were log-transformed to improve model fit [26]. For both tracers, we estimated varying (random) intercepts for the subjects and ROIs, and varying (random) slopes across ROIs for the predictor of interest (e.g., Total DEBQ score) and age. Compared to a model where the regionally specific effects would be estimated using interaction term for ROI, the hierarchical model produces results that are partially pooled toward each other, thus accounting for the multiple comparisons performed [60]. For both tracers, we also estimated regionally varying (random) residual variances. For [^11^C]carfentanil data, we also estimated regionally varying (random) intercepts for the scanners. We used the standard normal distribution as a prior distribution for the regression coefficients of the predictors to provide regularization. The standard normal distribution was also used as the prior distribution for the standard deviation of the group-level (random) effects. Otherwise we used the default priors of brms. We used 1000 warmup samples, 1000 post-warmup samples and 10 chains, thus totaling 10,000 post-warmup samples. The sampling parameters were slightly modified to facilitate convergence (*adapt_delta* = 0.999; *max_treedepth* = 20). The samplings produced no divergent iterations and the Rhats were all 1.0, suggesting that the chains converged successfully.

To examine associations in the whole brain, we used nonparametric approach with SnPM13 (http://nisox.org/Software/SnPM13/) to create full-volume linear regression models for *BP*_ND_ and *V*_T_ values. We used *p* < 0.01 as the cluster-defining threshold, and only report clusters large enough to be statistically significant at FWE *p* < 0.05. 5000 permutations were used to estimate the null distribution. We created distinct models for Total DEBQ score and all the subscale scores, adjusting for age and also for PET scanner in [^11^C]carfentanil models. The PET scanner was entered in the models as a covariate. Based on our earlier large-scale [^11^C]carfentanil data analysis, BMI in the current study range (18–31) is not associated with MOR availability [26]. However, to rule out the possible effects of sex, smoking and also BMI, we replicated the [^11^C]carfentanil full volume analysis with these additional covariates. The [^18^F]FMPEP-*d*_*2*_ models were also replicated with BMI as additional covariate (there were no smokers or females in the [^18^F]FMPEP-*d*_*2*_ data).

## Results

**Fig. 1:**
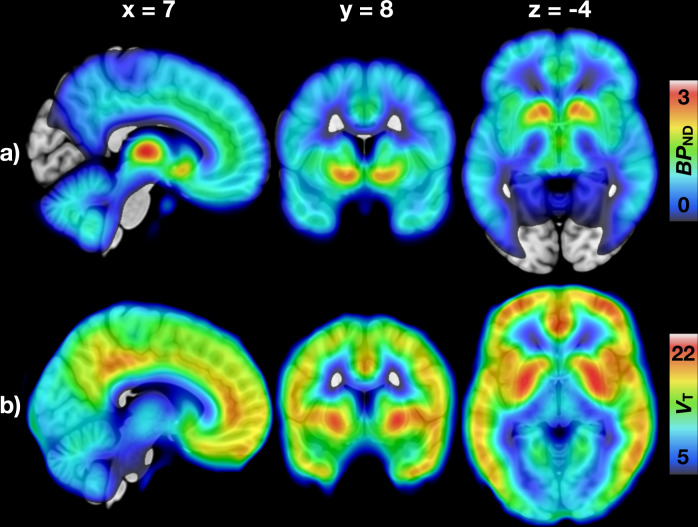
Mean distribution of central μ-opioid and CB_1_ receptors. **a** Mean binding potential (*BP*_ND_) of the 92 subjects (70 males and 22 females) studied with [^11^C]carfentanil. **b** Mean volume of distribution (*V*_T_) of the 35 males studied with [^18^F]FMPEP-*d*_*2*_.

Mean distribution of MORs and CB_1_Rs (Fig. 1) was consistent with previous studies [26, 50, 53, 61]. Correlations between the DEBQ subscales were positive but only modest, strongest being between Emotional and External eating (*r* = +0.33). BMI had a significant correlation only with Restrained eating (*r* = +0.27). Correlations with *p* values are presented in Supplementary Fig. 1 and Supplementary Table 3.

### Regional analysis of neuroreceptor availability and eating behavior

**Fig. 2:**
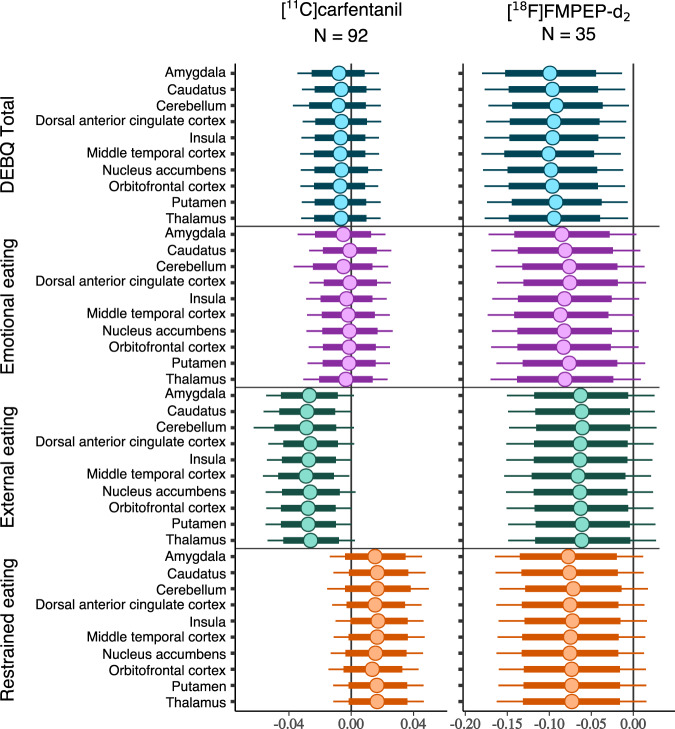
Regional associations of Total DEBQ and subscale scores with μ-opioid and CB_1_ receptor availabilities. The figure shows posterior distributions of the regression coefficients for Total DEBQ and subscale scores on log-transformed [^11^C]carfentanil binding potential (*BP*_ND_) and [^18^F]FMPEP-*d*_*2*_ volume of distribution (*V*_T_) in ten regions of interest. Age (and PET scanner for [^11^C]carfentanil data) are controlled as covariates. The colored circles represent posterior means, the thick horizontal bars 80% posterior intervals, and the thin horizontal bars 95% posterior intervals.

Higher External eating score was associated with lower [^11^C]carfentanil *BP*_ND_ in all a priori ROIs (Fig. 2). In the [^11^C]carfentanil models with other DEBQ subscales and Total DEBQ, the 80% confidence intervals overlapped with zero. For [^18^F]FMPEP-*d*_*2*_, higher Total DEBQ score was associated with lower *V*_T_ all examined ROIs (Fig. 2). The association directions between *V*_T_ and all DEBQ subscales were negative, but the 95% confidence intervals overlapped with zero. Complementary visualization of the regional relationships between DEBQ scores and neuroreceptor availability in representative ROIs is presented in Supplementary Fig. 2. In the subsample of 35 males with both [^11^C]carfentanil and [^18^F]FMPEP-*d*_*2*_ PET data, there were no significant regional correlations between MOR and CB_1_R availabilities (Supplementary Fig. 3).

### Full volume analysis of central receptor availability and eating behavior

For both tracers, full volume results were consistent with the ROI models. Mean receptor distribution maps and statistically significant DEBQ association maps can be found at NeuroVault (https://neurovault.org/collections/RZFLYXTL/).

#### μ-opioid receptor availability and DEBQ

**Fig. 3:**
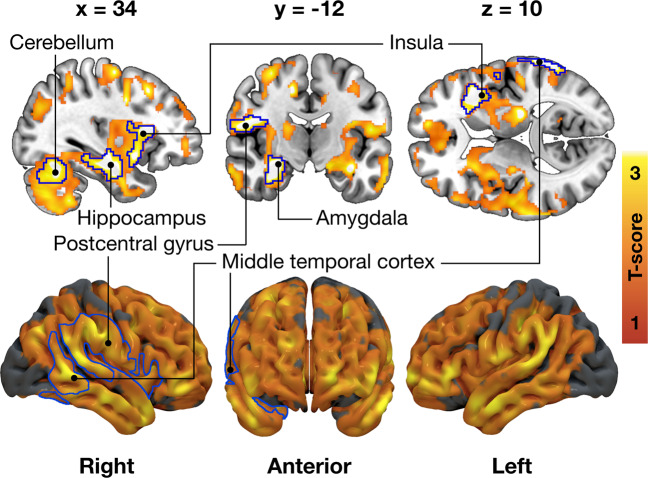
Association between External eating and decreased μ-opioid receptor availability in the 92 subjects (70 males and 22 females) scanned with [^11^C]carfentanil. The blue outline marks brain regions where lower [^11^C]carfentanil binding potential (*BP*_ND_) associated with higher External eating score, age and PET scanner as nuisance covariates, cluster forming threshold *p* < 0.01, FWE corrected. In the red–yellow T-score scale shown are also additional bilateral associations significant with more lenient cluster-defining threshold (*p* < 0.05, FWE corrected) for visualization purposes.

Higher External eating score was associated with lower [^11^C]carfentanil *BP*_ND_ in multiple brain areas (Fig. 3). Strongest cerebral associations were found in the right frontotemporal cortex and insula (peak voxel coordinates in Supplementary Table 4). Associations with Total DEBQ or other subscale scores were not statistically significant. Complementary analyses of [^11^C]carfentanil are presented in Supplementary Text 2 and Supplementary Fig. 4. In general, results were similar when additionally controlling for smoking, sex, and BMI. There were no significant associations in the female subsample, likely due to limited statistical power.

#### CB_1_ receptor availability and DEBQ

**Fig. 4:**
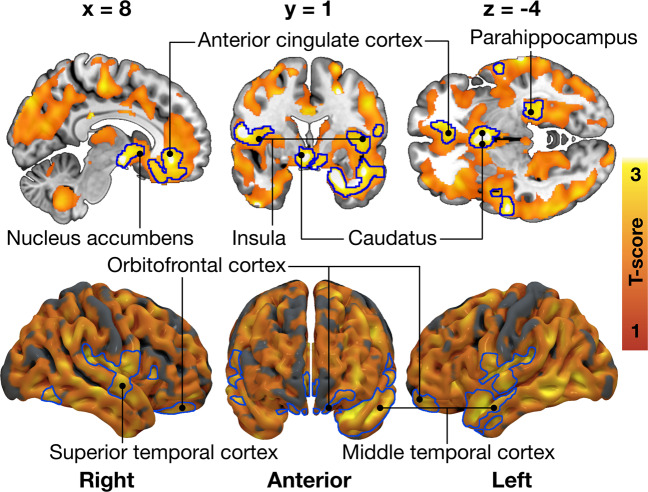
Total Dutch Eating Behavior Questionnaire (DEBQ) score associated with decreased CB_1_ receptor availability in the 35 males scanned with [^18^F]FMPEP-*d*_*2*_. The blue outline marks brain regions where lower [^18^F]FMPEP-*d*_*2*_ volume of distribution (*V*_T_) associated with higher Total DEBQ score, age as a nuisance covariate, cluster forming threshold *p* < 0.01, FWE corrected. In the red–yellow T-score scale shown are also additional associations significant with more lenient cluster-defining threshold (*p* < 0.05, FWE corrected) for visualization purposes.

Higher Total DEBQ score was associated with lower [^18^F]FMPEP-*d*_*2*_ *V*_T_ bilaterally in multiple brain regions (Fig. 4). Most prominent associations were found in parahippocampus, frontal striatum, insula, anterior cingulate, and frontotemporal cortices (peak voxel coordinates in Supplementary Table 4). Full-volume associations with distinct DEBQ subscales and *V*_T_ were not statistically significant. Results were essentially the same when controlling for BMI.

## Discussion

Our main finding was that higher DEBQ scores were associated with lower central availability of MORs and CB_1_Rs in healthy, nonobese humans. MOR and CB_1_R systems however showed distinct patterns of associations with specific dimensions of self-reported eating: While CB_1_Rs were associated in general negatively with different DEBQ subscale scores (and most saliently with the Total DEBQ score), MORs were specifically and negatively associated with externally driven eating only. Our results support the view that variation in endogenous opioid and endocannabinoid systems explain interindividual variation in feeding, with distinct effects on eating behavior measured with DEBQ.

### Central μ-opioid receptors and external eating behavior

External eating—the tendency to feed when encountering palatable food cues such as advertisements—was associated with lowered MOR availability in multiple brain areas, including insula, cortico-limbic regions and striatum, which are major areas processing environmental food cues and mediating reward [62]. A bulk of studies have shown that these regions are activated by mere perception of food cues or anticipation of feeding [63–65], and our recent work shows that lowered MOR availability is associated with amplified hemodynamic responses to food images in the same regions [15]. Higher score on external eating is associated with increased food craving [66] and cue-induced palatable food intake [38, 67], and may also contribute to short-term weight gain [40]. Altogether these results suggest that central MOR system has an important role in modulating particularly this kind of impulsive feeding that may lead to overweight.

Previous PET studies have established that feeding triggers endogenous opioid release in humans [22, 23]. Binge eating disorder (BED) is accompanied with downregulated central MORs and high External and Emotional eating scores [68]. Morbid obesity is also associated with lowered central MOR availability [23, 24], possibly reflecting receptor downregulation due to repeated overstimulation following feeding. In minipigs, already 12 days of high sucrose intake and following central endogenous neurotransmitter release downregulates MORs in cingulate and prefrontal cortices, nucleus accumbens and elsewhere in striatum [69]. The present findings extend the role of MORs in obesity and eating disorders to different feeding patterns in healthy subjects.

Healthy humans vary considerably in central MOR availability [26], and it is also possible that lowered MOR availability constitutes a genetically determined [70] risk factor for externally driven eating behavior. In healthy humans, trait impulsivity is associated with central MOR availability [27]. Increased cue-reactivity is prevalent feature of behavioral addictions [71], and patients with BED and pathological gambling have reduced availability of central MORs as measured with in vivo PET [54]. It is thus possible that subjects with lower MOR availability are susceptible for increased external eating in modern environment where they are consistently bombarded with feeding cues in advertisements and food shelves in supermarkets [11]. However, the present data are purely cross-sectional and longitudinal human studies are needed to further disentangle the causes and the effects between the decrease of MORs in relation to external eating.

The association with decreased MORs and high External eating scores was observed in all a priori ROIs. This was partly expected, since it is known that MOR densities exhibit high regional autocorrelation [72], and also in morbidly obese subjects, MOR availability is decreased globally [24]. These data suggest that the mechanism leading to decreased MOR availability affects the brain in a widespread manner. Preclinical research has found that exogenous MOR-agonists stimulate feeding [20], and in obese mice, central concentration of endogenous MOR-agonist (beta-endorphin) is increased manifold compared to controls [73]. Thus, one mechanism potentially leading to excessive external eating and compensatory MOR downregulation [74] could be chronically elevated basal endogenous opioidergic tone. Accordingly, the only available anti-obesity drug directly targeting opioid pathways is a combination of naloxone/bupropion, which blocks central beta-endorphin messaging and leads to stimulation of anorexigenic pathways [75, 76]. Although leading to 5–10% weight loss on average, the drug has major side effects [75]. Thus, it might be important to utilize also other types of pharmacological strategies. For example, in other conditions with chronically elevated opioidergic tone (such as tolerance following opioid abuse), ultra-low dose antagonists have been used successfully to restore MOR-mediated analgesic messaging [77, 78]. Whether these strategies might be applicable also in the treatment of obesity and externally-oriented feeding behavior is to be examined in future studies.

### Central CB_1_ receptors and eating behavior

Higher Total DEBQ score associated with lower availability of central CB_1_Rs, and ROI-level modeling suggested a consistent negative association with all DEBQ subscales. Compared with the [^11^C]carfentanil model, wider posterior distributions reflect the uncertainty arising from smaller [^18^F]FMPEP-*d*_*2*_ sample size. Brain’s endocannabinoid system is a major homeostatic signaling system, with CB_1_Rs abundant in all known food intake regulating central regions [79]. In previous brain PET studies, similarly lowered CB_1_R availability has been associated with increased BMI [35, 80], while globally upregulated CB_1_Rs have been found in anorexia nervosa [81]. These opposite phenotypes on body adiposity spectrum could potentially result from corresponding alterations from CB_1_R-regulated food intake behaviors. Indeed, stimulation of CB_1_Rs by pharmacological agonists or endocannabinoids promotes food intake and amplifies the rewarding properties of feeding [82]. In contrast, antagonism of the CB_1_Rs by rimonabant (withdrawn anti-obesity drug, Acomplia) effectively reduces food intake and increases energy expenditure, but in many patients also induces psychiatric symptoms (e.g., depressive mood, suicidality, anxiety) [79]. Accordingly, the endocannabinoid system function has been connected to several other essential behavioral processes in addition to feeding (e.g., stress-coping, emotion regulation, pain perception) [83, 84]. Being this diverse and complex regulatory system, it may not be possible to pinpoint single distinct aspect of food intake behavior mediated by CB_1_Rs. Rather, our results add support to central CB_1_Rs role in regulation of multiple eating behavior traits, with implications on both homeostatic and hedonic feeding [85].

### Limitations and methodological considerations

The [^11^C]carfentanil data were pooled from three PET scanners, which may produce minor variance in outcome measures [59]. However, this was accounted for in the analyses by adding the PET scanner as a nuisance covariate to all full-volume and regional analyses; the chosen outcome metric (*BP*_ND_) is also robust against such variability. The sample studied with [^11^C]carfentanil consisted predominantly of males, and our statistical power was compromised for detecting potential sex differences. The male and female samples were not identical with regards of age and Restrained eating score, which is due to the limited availability of database subjects. The sex difference in the [^11^C]carfentanil dose pertains to the fact that more females compared to males were scanned with HRRT PET scanner, which requires higher tracer doses (Supplementary Table 1)—however, this was accounted for by controlling with the scanner in all analyses as described above. Also all subjects of the [^18^F]FMPEP-*d*_*2*_ subsample were males, and thus conclusions regarding CB_1_Rs might not be generalizable to females. Eating behavior was assessed by self-reports, rather than by direct observations. DEBQ has however been found to successfully identify clinically relevant eating styles [4, 5]. Our study had a cross-sectional design, and although we found evidence of eating behavior’s association with MOR and CB_1_R systems, whether these receptor systems’ alterations directly promote future weight gain is to be examined in longitudinal studies. Our study included subjects with the BMI 18–31, and the findings might not be applicable to severe obesity. However, previous human PET studies have established that morbid obesity and eating disorders characterized by increased BMI are associated with decreased availability of MORs [24, 54] and CB_1_Rs [35]. Additional studies have found that these clinical conditions are also associated with increased DEBQ scores [5, 68]. Our study shows that MORs and CB_1_Rs contribute to feeding behavior regulation in a wide BMI range and in both healthy and clinical populations. Finally, in a single PET scan it is not possible to determine the exact proportions for causal factors to the altered receptor availability, which could potentially be affected by changes in receptor density, affinity or endogenous ligand binding [74].

## Conclusions

Low cerebral MOR availability is associated with increased externally triggered eating behavior. Modern obesogenic environment may promote food consumption via engaging the opioidergic link of the reward circuit whose tone is linked with cue-reactive eating. Our study suggests that for individuals with aberrant external eating, MOR system might provide a feasible pharmacological target to combat weight gain. Central CB_1_Rs are in turn associated with multiple eating behavioral traits measured with DEBQ, consistent with endocannabinoid system’s role as a major homeostatic regulatory system at the intersection of feeding, emotion and behavior.

## Supplementary information

Supplementary Material

## Data Availability

The code for preprocessing of the PET data (Magia) is available at https://github.com/tkkarjal/magia.
