# Supplementary material for: Cerebral μ-opioid and CB1 receptor systems have distinct roles in human feeding behavior
Source: Transl Psychiatry. 2021 Aug 27;11:442. doi: 10.1038/s41398-021-01559-5 (PMC8397789; doi:10.1038/s41398-021-01559-5)
Supplement: Supplementary file 1 — Supplementary Material [file 41398_2021_1559_MOESM1_ESM.pdf]

Supplementary Material to

Cerebral μ-opioid and CB_1-receptor systems have distinct roles in human feeding behavior

Tatu Kantonen[1,2], MD, Tomi Karjalainen[1], DSc (Tech), Laura Pekkarinen[1,3], MD, Janne Isojärvi[1], MSc, Kari Kalliokoski[1], PhD, Valtteri Kaasinen[2,4], MD, PhD, Jussi Hirvonen[1,5], MD, PhD, Pirjo Nuutila[1,3], MD, PhD, and Lauri Nummenmaa[1,6], PhD

[1]*Turku PET Centre, University of Turku, Finland*

[2]*Clinical Neurosciences, University of Turku, Finland*

[3]*Department of Endocrinology, Turku University Hospital, Finland*

[4]*Neurocenter, Turku University Hospital, Finland*

[5]*Department of Radiology, University of Turku and Turku University Hospital, Finland*

[6]*Department of Psychology, University of Turku, Finland*

Supplementary Material

i. PET scanners and smoking status (Supplementary Table 1)

ii. References for the original studies (Supplementary Table 2)

iii. Table of the p-values for descriptive correlations (Supplementary Table 3)

iv. Peak voxel coordinates for the full volume associations (Supplementary Table 4)

v. Scan acquisition and modeling in the PROSPECT (Supplementary Text 1)

vi. Complementary [^{11}C]carfentanil analyses (Supplementary Text 2)

vii. Descriptive correlations of the sample (Supplementary Figure 1)

viii. Visualization of regional associations in three representative regions of interest (Supplementary Figure 2)

ix. Regional correlations between MOR and CB_1R availabilities (Supplementary Figure 3)

x. External eating and μ-opioid receptor availability in males (Supplementary Figure 4)

PET scanners and smoking status

Supplementary Table 1. The information about PET scanners and smoking status of the sample.

	Males (n = 70)	Females (n = 22)
PET scanner (n)		
High Resolution Research Tool (HRRT, Siemens Medical Solutions)	8	9
Discovery 690 PET/CT (GE Healthcare)	10	13
GE Discovery VCT PET/CT (GE Healthcare)	52	0
Smoking status (n)*		
Smoking	0	7
Nonsmoking	57	15
Unknown	13	0

* The 13 individuals whose smoking status was unknown were classified as nonsmokers because in Finland, less than 20 % of the adult population smoked cigarettes in 2000 (http://urn.fi/URN:NBN:fi-fe2018102938947). Results were similar in a parallel analysis where individuals with unknown smoking status were classified as smokers.

References for the original studies

Supplementary Table 2. The references for the original studies whose applicable healthy controls were included in the current study sample (n = 92 [^{11}C]carfentanil scans).

Majuri J *et al.* Dopamine and Opioid Neurotransmission in Behavioral Addictions: A Comparative PET Study in Pathological Gambling and Binge Eating. *Neuropsychopharmacology* 2017; **42**(5)**:** 1169-1177.

- 17 subjects (8 males and 9 females) scanned with High Resolution Research Tool (HRRT, Siemens Medical Solutions).

Tuulari JJ *et al.* Feeding Releases Endogenous Opioids in Humans. *J Neurosci* 2017; **37**(34)**:** 8284-8291.

- 10 male subjects scanned with Discovery 690 PET/CT (GE Healthcare).

Karlsson HK *et al.* Obesity is associated with decreased mu-opioid but unaltered dopamine D2 receptor availability in the brain. *J Neurosci* 2015; **35**(9)**:** 3959-3965.

- 13 female subjects scanned with Discovery 690 PET/CT (GE Healthcare).

Nummenmaa L *et al.* Brain Basis of Psychopathy in Criminal Offenders and General Population. *Cerebral Cortex* (in press), https://emotion.utu.fi/wp-content/uploads/2021/03/Nummenmaa_et_al_CC_21.pdf

- 13 male subjects scanned with GE Discovery VCT PET/CT (GE Healthcare).

Neuromolecular Risk Factors for Obesity (PROSPECT), ClinicalTrials.gov Identifier: NCT03106688. https://www.clinicaltrials.gov/ct2/show/NCT03106688?term=neuromolecular+risk+factors+for+obesity&draw=2&rank=1

- 39 male subjects scanned with GE Discovery VCT PET/CT (GE Healthcare). All 35 [^{18}F]FMPEP-d_2 scans were from this subsample and also scanned with GE Discovery VCT PET/CT.

Table of the p-values for descriptive correlations

Supplementary Table 3. The p-values for corresponding Pearson correlation coefficients in the sample of 92 subjects (70 males and 22 females) scanned with [^{11}C]carfentanil.

	DEBQ Total	DEBQ Emotional	DEBQ External	DEBQ Restrained	BMI	Age
**DEBQ Total**	1.000	<0.001	<0.001	<0.001	0.137	0.119
**DEBQ Emotional**	<0.001	1.000	0.001	0.028	0.753	0.822
**DEBQ External**	<0.001	0.001	1.000	0.267	0.728	0.676
**DEBQ Restrained**	<0.001	0.028	0.267	1.000	0.009	<0.001
**BMI**	0.137	0.753	0.728	0.009	1.000	0.352
**Age**	0.119	0.822	0.676	<0.001	0.352	1.000

Peak voxel coordinates for the full volume associations

Supplementary Table 4. Peak voxel coordinates for the full volume associations.

Negative association between [^{11}C]carfentanil BP_{ND} and External eating score (n = 92)

Brain region	MNI coordinates x	y	z	T-score
Right middle temporal gyrus	66	-48	6	4.32
Right postcentral gyrus	54	-10	18	3.85
Right anterior insula	34	20	12	3.85
Cerebellum	26	-66	-18	3.87
	-16	-60	-22	3.48
	26	-52	-18	3.39

Negative association between [^{18}F]FMPEP-d_2 V_T and Total DEBQ score (n = 35)

Brain region	MNI coordinates x	y	z	T-score
Left superior temporal gyrus	-33	8	-33	4.00
	-48	7	-19	3.70
Left parahippocampus	-21	1	-23	3.67
Right parahippocampus	30	-35	-17	3.63
Right caudate	20	-21	23	3.08
Right cerebellum	30	-32	-30	2.96
Left middle frontal gyrus	-23	57	-16	3.42
Anterior cingulate cortex	2	2	-7	3.21
Anterior caudate	-1	14	-3	3.18
Right insula	42	-33	20	3.42
Right precentral gyrus	56	0	13	3.39
Right insula	37	-12	16	3.24

Cluster forming threshold $p < 0.01$, FWE corrected at cluster level. Age and PET scanner as covariates in the [^{11}C]carfentanil model, age as a covariate in the [^{18}F]FMPEP-d_2 model.

Scan acquisition and modeling in the PROSPECT

Supplementary Text 1. The acquisition and modeling of the 35 [^{18}F]FMPEP-d_2 scans and 39 [^{11}C]carfentanil scans from the PROSPECT project.

The 35 [^{18}F]FMPEP-d_2 scans and 39 [^{11}C]carfentanil scans are derived from the project "Neuromolecular Risk Factors for Obesity" (PROSPECT), which is preregistered to ClinicalTrials.gov (Identifier: NCT03106688). Subjects fasted at least 6 hours before the scans. The PET scans were done on separate days.

[^{18}F]FMPEP-d_2 scans

Two cannulas were inserted before the scan, one in an antecubital vein for [^{18}F]FMPEP-d_2 injection and one in the contralateral antecubital vein for blood sampling. Before the scan, venous blood samples were drawn to measure hematocrit. 147–215 MBq of [^{18}F]FMPEP-d_2 was administered intravenously and brain's radioactivity was followed 60 minutes (15 frames). A peripheral PET scan was conducted on neck (12 min) and abdominal (9 min) regions. Brain's radioactivity was then followed 9 minutes (3 x 180-second frames) starting at a time point 94 ± 2 min. Arterialized venous blood samples to measure plasma activity were drawn contralaterally to injection site at 0.25, 0.5, 0.75, 1, 1.25, 1.5, 1.75, 2, 2.5, 3, 4.5, 7.5, 11, 15, 20, 25, 30, 35, 40, 45, 50 and 60 min from the injection, and at subsequent time points (min from the regional scan start): neck (2 and 6 min), abdomen (4.5 min) and late brain scan (4.5 min). Additional blood samples for [^{18}F]FMPEP-d_2 metabolite analysis were drawn before the scan and at 4.5, 11, 15, 20, 30, 45 and 60 min from the injection, and at subsequent time points (min from the regional scan start): neck (2 and 6 min), abdomen (4.5 min).

CB_1R availability was quantified as [^{18}F]FMPEP-d_2 volume of distribution (V_T) using graphical analysis[1] (Logan). The frames starting at 36 minutes and later since injection were used in the model fitting, since Logan plots became linear after 36 minutes[1]. Plasma activities were

corrected for plasma metabolites as described previously[2]. We were unable to obtain metabolite data from two subjects due to laboratory measurement issues, so we used the mean metabolite fraction of the group for these two subjects.

[^{11}C]carfentanil scans

A cannula was inserted before the scan in an antecubital vein for [^{11}C]carfentanil injection. 223–279 MBq of [^{11}C]carfentanil was administered intravenously and the brain's radioactivity was followed 51 minutes (13 frames). The modeling of the [^{11}C]carfentanil data and the acquisition of binding potentials (BP_{ND}) has been previously described[3].

Complementary [^{11}C]carfentanil analyses

Supplementary Text 2. Summary of the complementary [^{11}C]carfentanil analyses.

Smoking status did not influence [^{11}C]carfentanil results. When adjusting simultaneously for smoking, sex and BMI (in addition to age and scanner), the association with [^{11}C]carfentanil binding potential (BP_{ND}) and External eating score remained essentially the same (significant at cluster forming threshold $p < 0.02$, FWE corrected).

The male subsample

In the subsample of 70 males, the associations between External eating score and μ-opioid receptor availability were mostly similar than with the full sample, associations in caudatus being slightly less prominent (significant at cluster forming threshold $p < 0.05$, FWE corrected).

The female subsample

In the subsample of 22 females, there were no statistically significant associations between External eating score and central μ-opioid receptor availability. This is potentially due to the relatively low number of female subjects.

Descriptive correlations of the sample

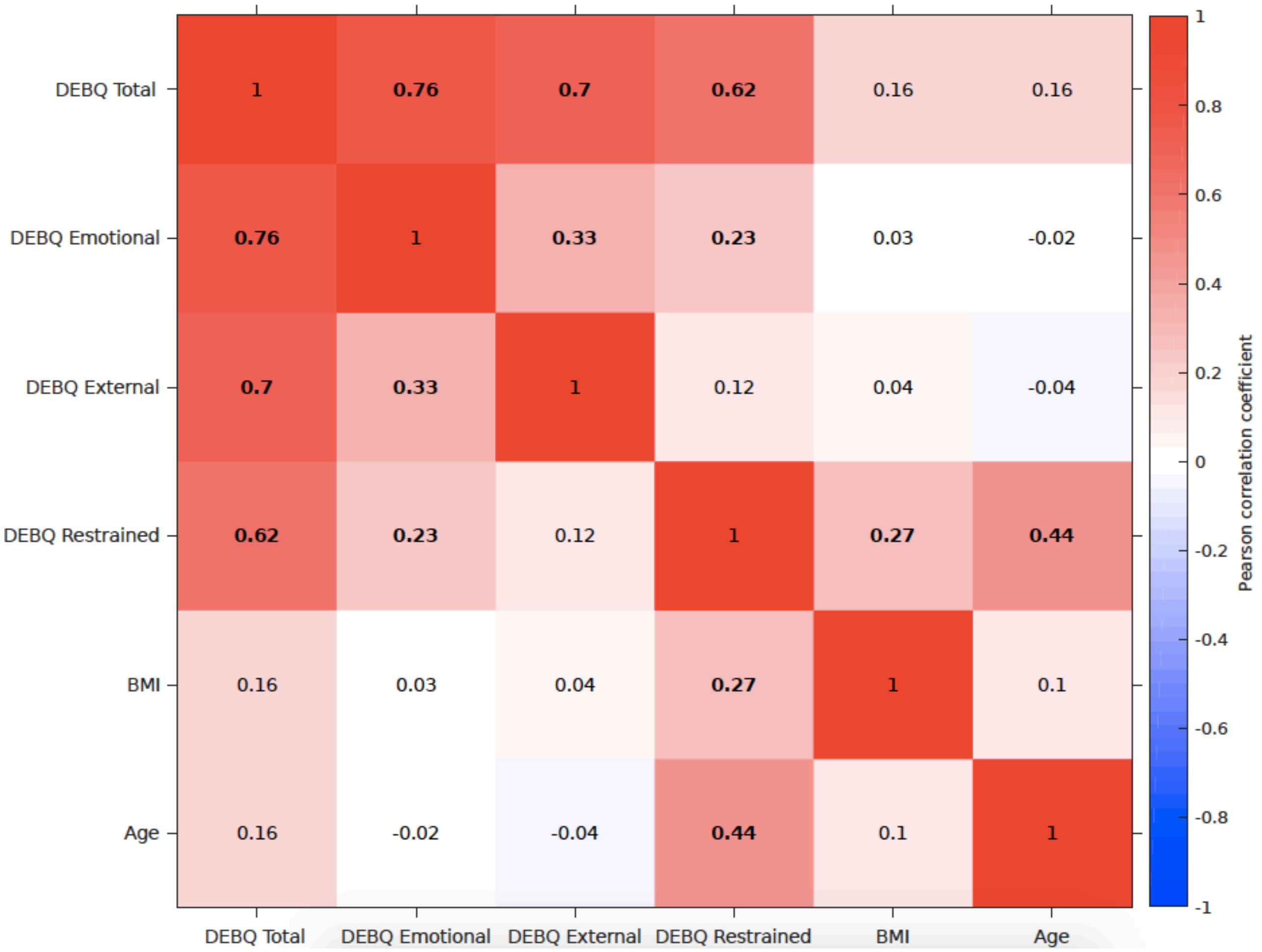

Supplementary Figure 1. Descriptive correlations between the Dutch Eating Behaviour Questionnaire (DEBQ) total and subscale scores, age and body mass index (BMI) in the sample of 92 subjects (70 males and 22 females) scanned with [^{11}C]carfentanil. The numbers indicate Pearson correlation coefficients. $p < 0.05$ correlations are marked with boldface.

Since Restrained eating score was higher in females than in males and females were also older than males (**Table 1**), we examined the correlations of age and restrained eating also in both female and male subgroups. Restrained eating correlated positively with age in both groups, but more strongly in the female sample (females $r = 0.33$, $p = 0.01$; males $r = 0.25$, $p = 0.03$).

Visualization of regional associations in three representative regions of interest

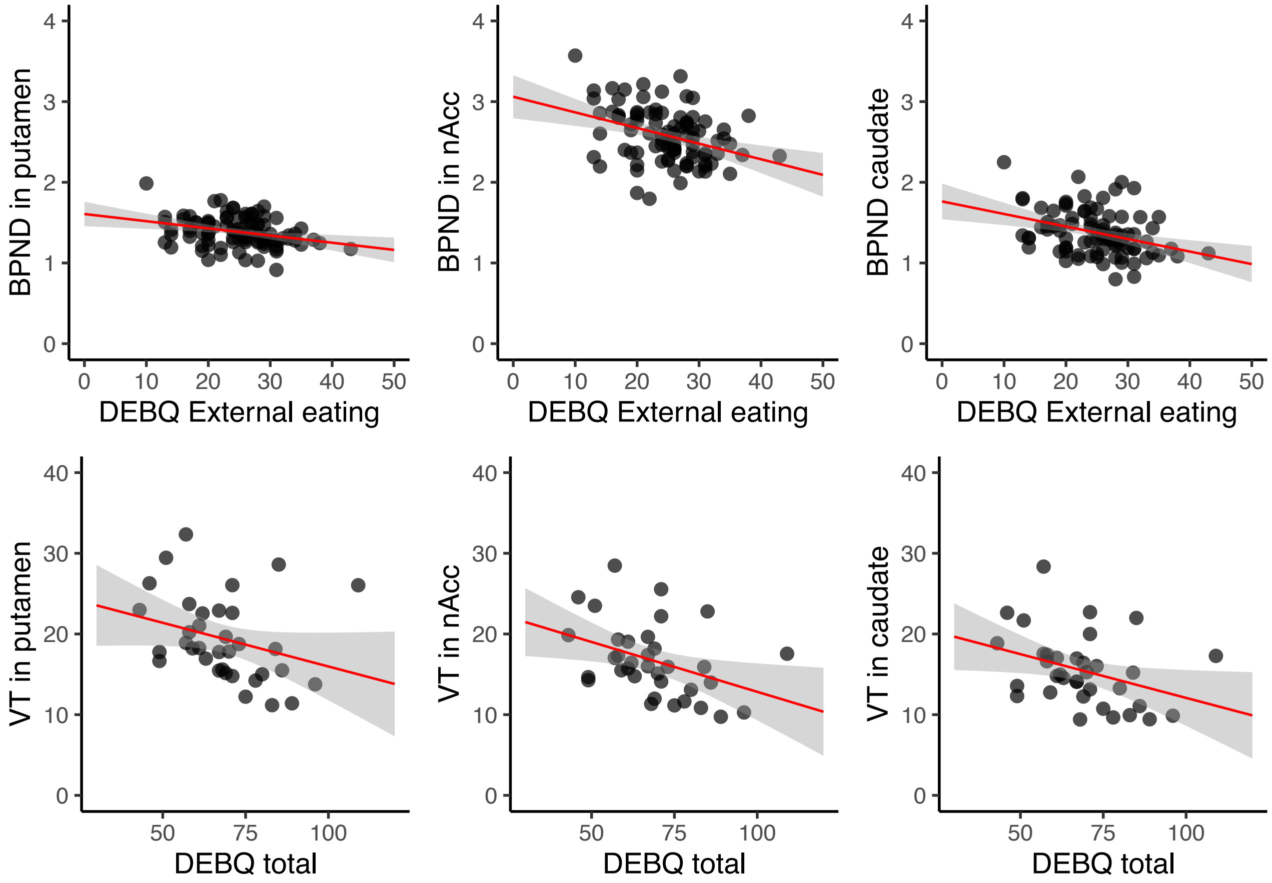

Supplementary Figure 2. Visualization of regional associations in three representative regions of interest. Upper row: Scatterplots show the associations of External eating score and [^{11}C]carfentanil binding potential (BP_{ND}) in putamen, nucleus accumbens (nAcc) and caudate (92 subjects, LS-regression line with 95% CI). Lower row: Scatterplots show the associations of Total DEBQ score and [^{18}F]FMPEP-d_2 volume of distribution (V_{T}) in putamen, nucleus accumbens (nAcc) and caudate (35 subjects, LS-regression line with 95% CI).

Regional correlations between MOR and CB_1R availabilities

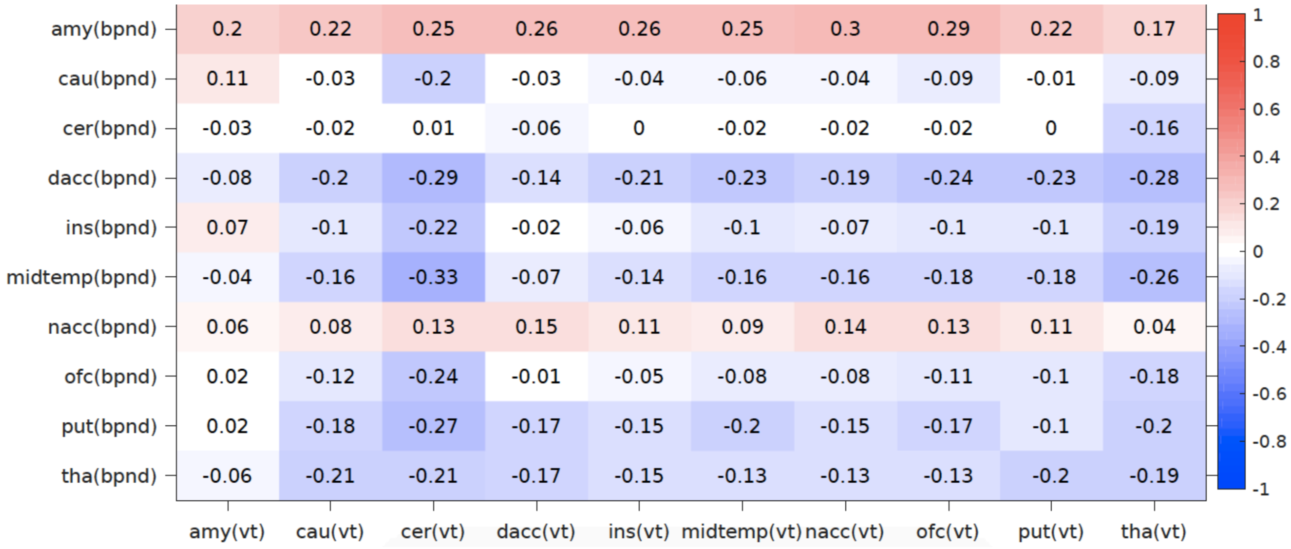

Supplementary Figure 3. Descriptive correlations between [^{11}C]carfentanil binding potential BP_{ND} (bpnd) and [^{18}F]FMPEP-d_2 volume of distribution V_{T} (vt) in 10 regions of interest in the subsample of 35 subjects scanned with both tracers. The numbers indicate Pearson correlation coefficients (for all, $p > 0.05$). The abbreviations stand for amy = amygdala, cau = caudatus, cer = cerebellum, dacc = dorsal anterior cingulate cortex, ins = insula, midtemp = middle temporal cortex, nacc = nucleus accumbens, ofc = orbitofrontal cortex, put = putamen, tha = thalamus.

External eating and μ-opioid receptor availability in males

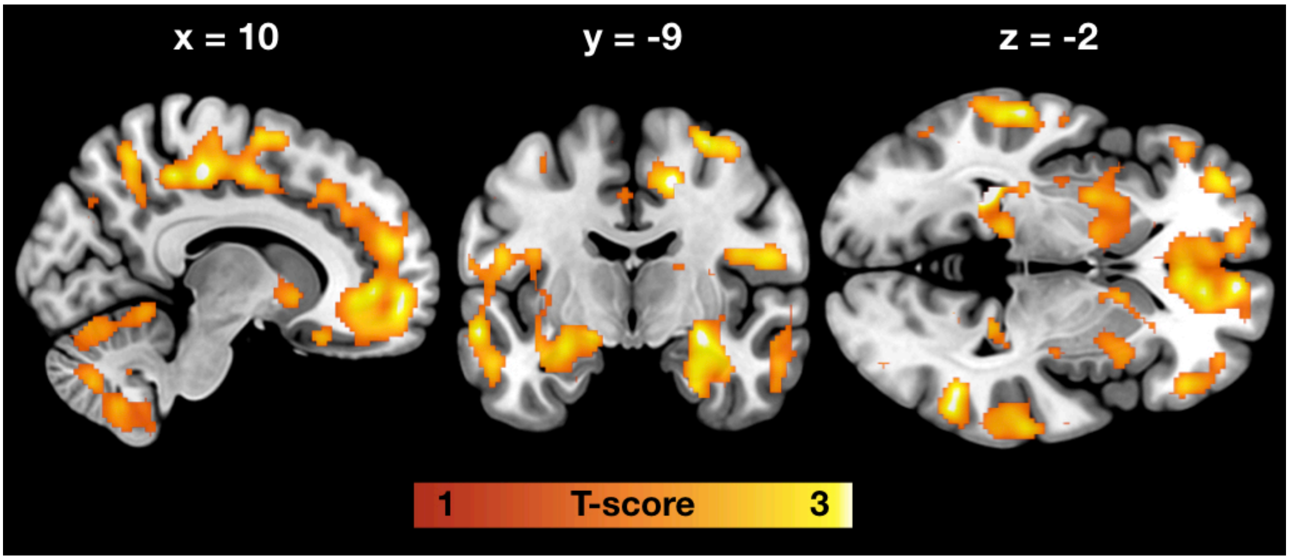

Supplementary Figure 4. Brain regions where higher External eating score associated with lower [^{11}C]carfentanil binding potential (BP_{ND}) in 70 males, age and PET scanner as nuisance covariates. Shown are clusters where $p < 0.05$, FWE corrected at cluster level.

References

1. Logan J. Graphical analysis of PET data applied to reversible and irreversible tracers. *Nuclear Medicine and Biology* 2000; **27**(7)**:** 661-670.

2. Lahesmaa M *et al.* Cannabinoid type 1 receptors are upregulated during acute activation of brown adipose tissue. *Diabetes* 2018; **67**(7)**:** 1226-1236.

3. Kantonen T *et al.* Interindividual variability and lateralization of μ-opioid receptors in the human brain. *NeuroImage* 2020; **217:** 116922.
